# Protura of Italy, with a key to species and their distribution

**DOI:** 10.3897/zookeys.146.1885

**Published:** 2011-11-09

**Authors:** Loris Galli, Matteo Capurro, Carlo Torti

**Affiliations:** 1 Dipartimento per lo studio del Territorio e delle sue Risorse, Università degli Studi di Genova, Corso Europa 26, I-16132 Genova, Italy

**Keywords:** Protura, Italy, distribution, key to species

## Abstract

The Italian Protura were studied basing on 5103 specimens from 198 sampling areas, along with bibliographic data from 49 collecting sites. 17 out of the 20 Italian regions are covered. As a result, 40 species have been identified (belonging to 8 genera and 4 families), 6 of which are new records for the Italian fauna.

A key to the Italian species is reported, followed by a series of distribution maps and brief remarks for some of them. A preliminary biogeographical overview allowed us to delineate the chorological categories of these species, 10 of which are actually known only in Italy. The comparison with the species richness known for some best studied Central and Eastern European Countries leads us to speculate that widening our research, Italian Protura check-list will be much implemented.

## Introduction

Protura is a group of Hexapoda which has been discovered recently: the first species described is *Acerentomon doderoi*, collected from soil samples taken from the grounds of a small villa actually in the center of Genoa (Silvestri 1907). More detailed data about such taxon are provided in the two years immediately following by Berlese (1908a, 1908b, 1909).

Knowledge of Protura has rapidly increased all over the world thanks to the careful research of many specialists. Just to mention the main publications, in 1964 Tuxen published his valuable book about the Protura of the World; Nosek’s monograph on European Protura was printed in 1973; a year later Imadaté’s volume about Japanese species was released (1974); while the impressive monograph about Chinese Protura was published more recently (Yin 1999).

The European research about this group, although with some exceptions, was concentrated in Central and Eastern Countries due to the work of some Authors such as Nosek, Rusek, Tuxen and, more recently, Szeptycki and Shrubovych.

In Italy, knowledge regarding Protura (see species list for detailed bibliographic references) can be summarized with the identification of 31 species belonging to the Italian fauna by the national check-list (Dallai et al. 1995). The same number of species emerges by an overview of the most recent Catalogue of the World Protura (Szeptycki 2007).

With this paper we hope to lay the foundations for the advancement and improvement of studies regarding this little known taxon in Italy as well in the Mediterranean Region, one of the biodiversity hotspots on the planet, reaching the highest peaks of diversity of soil-borne organisms (e.g. Blondel et al. 2010).

## Materials and methods

Many of the Protura examined in this paper were collected by colleagues and given us in tubes containing 70% ethanol. However, we have obtained some specimens by extraction from soil or litter samples by Berlese-Tullgren funnels (2.5 mm mesh size). Specimens were incubated at 40–50° C for 24 hours in lactic acid to clarify, mounted on slides in Marc André medium and were observed and identified by an interference contrast microscope.

In total 5103 specimens from 198 sampling areas were examined. 3929 specimens were identified to species level (Table 1).

**Table 1.** Number of Protura specimens examined from each Italian region.

**Regions**	**Nr of specimens (bibliographic data excluded)**	**Nr of specimens identifiable to species level (bibliographic data excluded)**
Aosta Valley	134	94
Piedmont	748	546
Lombardy	214	189
Trentino-Alto Adige	40	20
Veneto	193	161
Friuli-Venezia Giulia	68	58
Liguria	2878	2158
Emilia-Romagna	149	143
Tuscany	264	228
Marches	3	2
Umbria	45	46
Lazio	64	51
Abruzzo	22	16
Molise	-	-
Campania	-	-
Apulia	16	7
Basilicata	67	45
Calabria	-	-
Sicily	33	26
Sardinia	165	139
**Total**	**5103**	**3929**

In our analysis we also considered the data taken from 49 Italian collecting sites known in literature. 17 out of 20 Italian regions are covered, missing specimens from Molise, Campania and Calabria (Southern part of the peninsula).

### Key to genera of Italian Protura

This key and the following ones to species are based, and adapted to the Italian fauna, on Nosek (1973) and Szeptycki (1980, 1985, 1986, 1991).

**Table:** 

1	Tracheal system present (meso and metanotum with spiracles); all three pairs of abdominal legs two segmented, with terminal vescicle and with 5 setae on each	Eosentomidae – Genus *Eosentomon*
–	Spiracles absent	Acerentomoidea 2
2	Only the first pair of abdominal legs with a terminal vescicle and 4 setae; pairs II and III unsegmented with 2–3 setae; abdominal segment VIII with a more or less developed striate band	Acerentomidae 4
–	First two pairs of abdominal legs with terminal vescicle; third pair unsegmented	3
3	Maxillary gland with a long dilatated sausage–like part; pseudoculus pear–like with a long and broad S shaped median opening; 8 setae in the anterior row of abdominal tergites II–VII	Hesperentomidae – *Ionescuellum condei* (Nosek, 1965)
–	At most 4 setae in the anterior row of abdominal tergites II–VII; maxillary gland with heart–shaped or circular dilatation; pseudoculi without median opening	Protentomidae 7
4	Abdominal legs II and III with 3 setae (a longer median one and two shorter sub–apical)	Genus *Acerentulus*
–	Abdominal legs II and III with 2 setae	5
5	Abdominal legs II and III with 2 setae of the same length; maxillary gland with racemose appendix; sensillum of labial palp broad	Genus *Acerella*
–	Subapical seta of abdominal legs II and III shorter than the median one	6
6	Abdominal legs II and III with a long median seta and a very short subapical one; pseudoculi small; striate band of tergite VIII complete; maxillary gland with a rather large calyx, heart shaped; tuft of setae on labial palp strongly reduced; anterior row of abdominal sternites I–VII with 3 setae; sternite VIII with a single row of 4 setae	Genus *Gracilentulus*
–	Subapical seta of abdominal legs II and III half the length of the median one or less; head with a rostrum (from very short to long); anterior row of abdominal sternites I–VII with a variable number (≥ 3) of setae	Genus *Acerentomon*
7	Pseudoculus with a large triangular proximal prolongation; the “lever” of the same length as the pseudoculus itself and of almost the same width distally; the comb on tergite VIII with distinct teeth	Genus *Proturentomon*
–	Pseudoculus often more elliptical and proximal prolongation usually narrower parallel sided; the comb on tergite VIII with very fine teeth or toothless	Genus *Protentomon*

### Keys to species of Italian Protura

Since this key could lead to a misidentification of similar Palearctic species not already detected in Italy, we suggest a careful examination of the species’ descriptions (and redescriptions) to verify the identification accuracy and also to refer to the keys to species of other European Countries (e.g. those cited at the beginning of the key to genera) as well as to the monographic papers published on certain genera (e.g. Rusek 1975; Szeptycki 1993).

**Genus *Eosentomon***

**Table:** 

1	Tergite VII with 6 anterior setae	2
–	Tergite VII with 4 anterior setae	3
2	Head with only posterior additional seta; seta p2’ on nota shorter than p3’	*Eosentomon transitorium* Berlese, 1909
–	Head with only posterior additional seta; seta p2’ roughly the same length of p3’	*Eosentomon germanicum* Prell, 1912
3	Tergites IV–VI missing seta p4’; chaetotaxy of sternite XII 8/7	*Eosentomon foroiuliense* Torti & Nosek, 1984
–	Tergites IV–VI with seta p4’; chaetotaxy of sternite XII 8/4	4
4	Tergites II–VI missing seta p3’	*Eosentomon romanum* Nosek, 1969
–	Tergites II–VI with seta p3’; head with both anterior and posterior additional setae; seta p2’ on nota subequal or longer than p3’	5
5	On tergite VII seta p1’ situated at the same level and near the base of p2	*Eosentomon delicatum* Gisin, 1945
–	On tergite VII seta p1’ placed close to the posterior border and p2’ in a cavity on the hind margin	6
6	Sensillum c’ behind the line α6–δ5; body length 750 μm; pseudoculus fairly big (PR = 7.5)	*Eosentomon noseki* Tuxen, 1982
–	Sensillum c’ proximally to line α6–δ5; body length 1610 μm; PR = 8.6–11.6	*Eosentomon armatum* Stach, 1926

**Genus *Acerentulus***

**Table:** 

1	Sensillum a long reaching nearly or passing seta γ3; sensillum b subequal or shorter than c	4
–	Sensillum a of medium length or short, not reaching or barely reaching seta γ3	2
2	Sensillum b subequal or shorter than c	3
–	Sensillum b much longer than c, reaching the empodium	*Acerentulus traegardhi* Ionescu, 1937
3	Tergites II–VI without seta p1’	*Acerentulus cunhai* Condé, 1950
–	Tergites II–VI with seta p1’	*Acerentulus tuxeni* Rusek, 1966
4	Sensillum a’ broad, relatively short, not reaching the base of b’	5
–	Sensillum a’ broad, long, reaching the base of b’	8
5	Seta p3’ missing in tergite VII	6
–	Seta p3’ present in tergite VII	7
6	Seta a1 missing in tergite VII	*Acerentulus exiguus* Condé, 1944
–	Seta a1 present in VII	*Acerentulus apuliacus* Rusek & Stumpp, 1988
7	Setae a1 and p1’ missing in tergite VII	*Acerentulus gisini* Condé, 1952
–	Setae a1 and p1’ present in tergite VII	*Acerentulus confinis* (Berlese, 1908)
8	Setae a1 and p1’ missing in tergite VII	9
–	Tergite VII: seta a1 present, p1’ missing	*Acerentulus terricola* Rusek, 1965
9	Seta p1 present in tergite VIII; sternite XI with 4 setae	*Acerentulus condei* Nosek, 1983
	Seta p1 missing in tergite VIII; sternite XI with 6 setae	*Acerentulus alpinus* Gisin, 1945

**Genus *Acerella***

**Table:** 

1	Sensillum t2 nearly 3 times the length of t1	*Acerella tiarnea* (Berlese, 1908)
–	Sensillum t2 one and a half to twice the length of t1	*Acerella muscorum* (Ionescu, 1930)

**Genus *Gracilentulus***

**Table:** 

1	Sensillum b not reaching seta γ3	2
–	Sensillum b passing seta γ3	*Gracilentulus gracilis* (Berlese, 1908)
2	Chaetotaxy of tergites II–VI 7/16; TR = 2,7	*Gracilentulus sardinianus* Nosek, 1979
–	Chaetotaxy of tergites II–VI 8/14; TR = 3,3	*Gracilentulus meridianus* (Condé, 1945)

**Genus *Acerentomon***

**Table:** 

1	Chaetotaxy of sternite VIII 4/0	2
–	Chaetotaxy of sternite VIII 4/2	3
2	Seta x present; rostrum very long, LR = 3,3	*Acerentomon noseki* Torti, 1981
–	Seta x absent; rostrum short, LR ≥ 6	4
3	Seta x present; rostrum long, LR = 3,5–4,7	7
–	Seta x absent; rostrum of medium length, LR = 4,5–5	*Acerentomon affine* Bagnall, 1912
4	Rostrum short, LR nearly 6	5
	Rostrum very short, LR ≥ 9	6
5	Sensillum b extremely broad, not spindle–shaped and shorter than c; a long and reaching the base of e; pleural pectines strongly developed	*Acerentomon meridionale* Nosek, 1960
–	Sensillum b broad, spear shaped, almost reaching seta γ4 and longer than c; a short, barely reaching the base of d; pleural pectines only on segments VI–VII	*Acerentomon balcanicum* Ionescu, 1933
6	Comb VIII with 10–14 teeth; pleural pectin VI with a row of long teeth; ratio of sensilla a:b = 1,1	*Acerentomon microrhinus* Berlese, 1909
–	Comb VIII with 17–20 teeth; pleural pectin VI strongly reduced to a group of 4 distinct teeth; ratio of sensilla a:b = 0,8	*Acerentomon condei* Nosek & Dallai, 1982
7	Sensillum b thin and small, distinctly shorter than c	8
–	Sensillum b distinctly broad, subequal or longer than c	10
8	Head with additional setae	*Acerentomon gallicum* Ionescu, 1933
–	Head without additional setae	9
9	Sensillum a short, barely reaching d; pleural line VI with a fine serration	*Acerentomon italicum* Nosek, 1969
–	Sensillum a long, extending beyond the base of d, sometimes even reaching e; pleural line VI with a row of conspicuous teeth	*Acerentomon fageticola* Rusek, 1966
10	Seta p3’ present in tergite VII	*Acerentomon doderoi* Silvestri, 1907
–	Seta p3’ missing in tergite VII	11
11	Comb VIII with 14–16 long teeth, the median ones smaller; body length about 1600 μm	*Acerentomon maius* Berlese, 1908
–	Comb VIII with 9–12 pointed teeth; body length 1980–2370 μm	*Acerentomon baldense* Torti, 1986

**Genus *Proturentomon***

**Table:** 

1	Chaetotaxy of tergites I–VI 0/12	*Proturentomon noseki* (Rusek, 1975)
–	Chaetotaxy of tergites II–VI 2/12	2
2	Chaetotaxy of tegites I and VIII 2/10 and 6/12, respectively; sensillum b distinctly shorter than c	*Proturentomon minimum* Berlese, 1908
–	Chaetotaxy of tegites I and VIII 2/12 and 6/14, respectively; sensillum b subequal or longer than c	3
3	Body length 690 μm; comb on tergite VIII with 8 teeth	*Proturentomon condei* Nosek, 1967
–	Body length 500 μm; comb on tergite VIII with 4 long and thin teeth	*Proturentomon pilosum* (Rusek, 1975)

**Genus *Protentomon***

**Table:** 

1	Tergites II–VI without the anterior row of setae; sternite XI with 4 setae	*Protentomon perpusillum* (Berlese, 1909)
–	Tergites II–VI with 2 setae in the anterior row; sternite XI with 6 setae	*Protentomon berlesei* Nosek, 1969

## Italian Protura

This section provides summaries on species known to date belonging to Italian fauna. For each one the amount of material examined (PI = pre-imago, MJ = maturus junior, LII = larva II, LI = larva I, undet = undetermined), a short description of the global distribution from Szeptycki (2007) and, when necessary, some remarks are given. For the new recorded Italian species more geographical details (locality, province and region) are given. The maps (Figs 1–25) show the collecting areas in Italy: blue dots represent collecting sites known only in literature, while the red ones correspond to samples personally analyzed by the authors.

### ORDO: ACERENTOMATA

#### Familia: Hesperentomidae Price, 1960

##### Ionescuellum condei

(Nosek, 1965)

http://species-id.net/wiki/Ionescuellum_condei

Fig. 1 

###### Material examined.

4 ♂♂, 4 ♀♀.

###### Distribution.

Austria, Italy.

**Figure 1.**
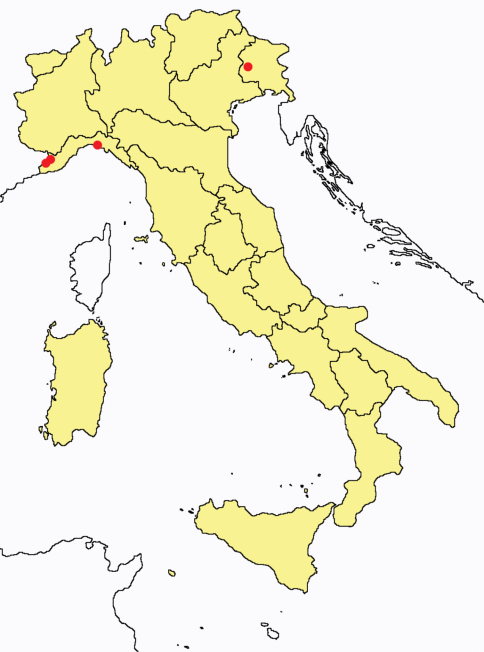
*Ionescuellum condei*: collecting sites in Italy (red dots: samples personally analyzed by the authors).

###### Remarks.

First Italian record in Torti (1981a). Some generic records from Lombardy (N Italy) of *Hesperentomon* sp. in (Dematteis (1971, 1972) could be attributed to this species.

#### Familia: Protentomidae Ewing, 1936

##### Protentomon berlesei

Nosek, 1969

http://species-id.net/wiki/Protentomon_berlesei

Fig. 2 

###### Material examined.

2 ♂♂, 2 ♀♀.

###### Type area.

Veneto, Colli Euganei near Padua.

###### Distribution.

Italy.

**Figure 2.**
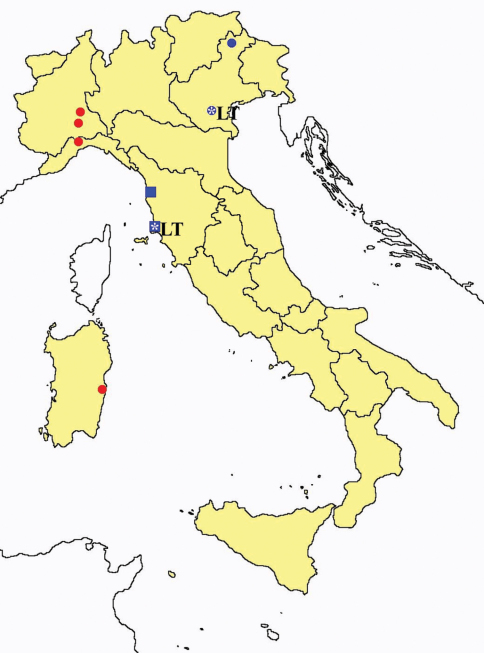
*Protentomon* spp.: collecting sites in Italy (dots *Protentomon berlesei*; squares: *Protentomon perpusillum*; blue: data from literature; red: samples personally analyzed by the authors; LT = type area).

###### Remarks.

For nearly 40 years since its description (Nosek 1969), only two specimens (holotype and paratype) from Veneto (NE Italy) belonging to this species were known. In 2007 two of us (Galli and Torti) published a short note about a third specimen from Liguria (NW). Three other specimens were most recently found in samples from Piedmont (NW) and Sardinia.

##### Protentomon perpusillum

(Berlese, 1909)

http://species-id.net/wiki/Protentomon_perpusillum

Fig. 2 

###### Type area.

Tuscany, S. Vincenzo (Livorno).

###### Distribution.

Italy, Germany. Data from Denmark and Australia should be confirmed (Szeptycki 2007).

###### Remarks.

Bibliographic data from Berlese (1909), Nosek (1973).

##### Proturentomon condei

Nosek, 1967

http://species-id.net/wiki/Proturentomon_condei

Fig. 3 

###### Material examined.

6 ♀♀, 1 MJ.

###### Distribution.

Austria, Slovakia.

**Figure 3.**
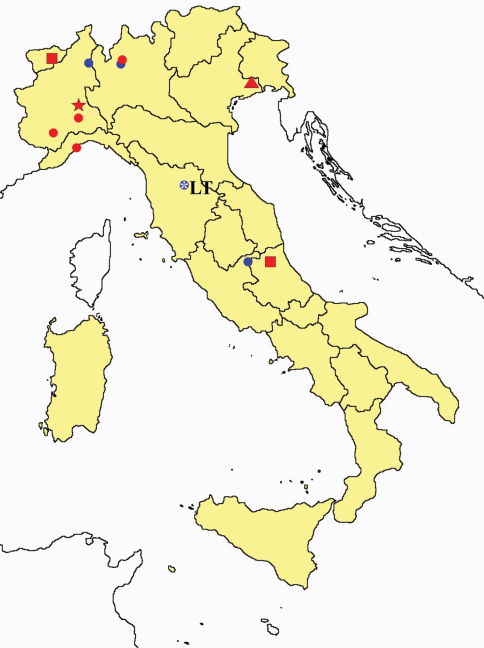
*Proturentomon* spp.: collecting sites in Italy (squares *Proturentomon condei*; dots *Proturentomon minimum*; star: *Proturentomon noseki*; triangle *Proturentomon pilosum*; blue: data from literature; red: samples personally analyzed by the authors; LT = type area).

###### Remarks.

This species is not included in the World Catalogue (Szeptycki 2007) because it was recorded in Italy only more recently (Capurro et al. 2008b).

##### Proturentomon minimum

(Berlese, 1908)

http://species-id.net/wiki/Proturentomon_minimum

Fig. 3 

###### Material examined.

11 ♀♀, 7 MJ, 1 undet.

###### Type area.

Tuscany, Giardino di Boboli in Florence.

###### Distribution.

Recorded from nearly whole Europe (with exception of Scandinavia), but all of the older data should be confirmed (Szeptycki 2007).

###### Remarks.

Bibliographic data from Berlese (1908), Dematteis (1971, 1972), Nosek (1973).

##### Proturentomon noseki

Rusek, 1975

http://species-id.net/wiki/Proturentomon_noseki

Fig. 3 

###### Material examined.

2 ♀♀ (Vignale Monferrato, Alessandria, Piedmont).

###### Distribution.

Central Europe.

###### Remarks.

New record for the Italian fauna.

##### Proturentomon pilosum

Rusek, 1975

http://species-id.net/wiki/Proturentomon_pilosum

Fig. 3 

###### Material examined.

1 ♀ (Concordia, Venice, Veneto).

###### Distribution.

Central Europe.

###### Remarks.

New record for the Italian fauna.

#### Familia: Acerentomidae Silvestri, 1907

##### Acerentulus alpinus

Gisin, 1945

http://species-id.net/wiki/Acerentulus_alpinus

Fig. 4 

###### Distribution.

South Europe.

**Figure 4.**
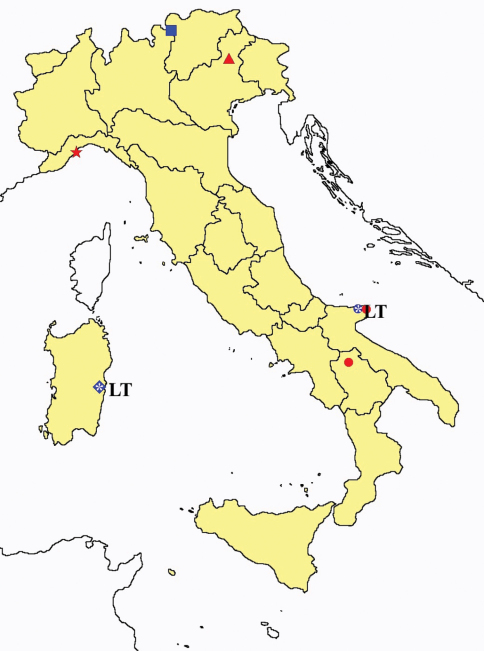
*Acerentulus* spp.: collecting sites in Italy (square: *Acerentulus alpinus*; dots: *Acerentulus apuliacus*; rhombus: *Acerentulus condei*; star: *Acerentulus terricola*; triangle: *Acerentulus tuxeni*; blue: data from literature; red: samples personally analyzed by the authors; LT = type area).

###### Remarks.

Bibliographic data from Dematteis (1972).

##### Acerentulus apuliacus

Rusek & Stumpp, 1988

http://species-id.net/wiki/Acerentulus_apuliacus

Fig. 4 

###### Material examined.

3 ♂♂, 20 ♀♀, 3 PI, 1 MJ.

###### Type area.

Apulia, 10 km South of Vico del Gargano, Bosco Sfilzi.

###### Distribution.

Type area only.

###### Remarks.

Bibliographic data from Rusek and Stumpp (1988).

##### Acerentulus condei

Nosek, 1983

http://species-id.net/wiki/Acerentulus_condei

Fig. 4 

###### Type area.

Sardinia, Strada Orientale Sarda km 158.

###### Distribution.

Mediterranean Europe (Sardinia, Corsica, Slovenia).

###### Remarks.

Bibliographic data from Nosek (1983).

##### Acerentulus confinis

(Berlese, 1908)

http://species-id.net/wiki/Acerentulus_confinis

Fig. 5 

###### Material examined.

104 ♂♂, 187 ♀♀, 5 PI, 24 MJ.

###### Type area.

Tuscany, Florence.

###### Distribution.

Recorded from nearly all Europe (with exception of Scandinavia), North Africa, North America and Australia. Most of the older data are dubious and should be confirmed (Szeptycki 2007).

**Figure 5.**
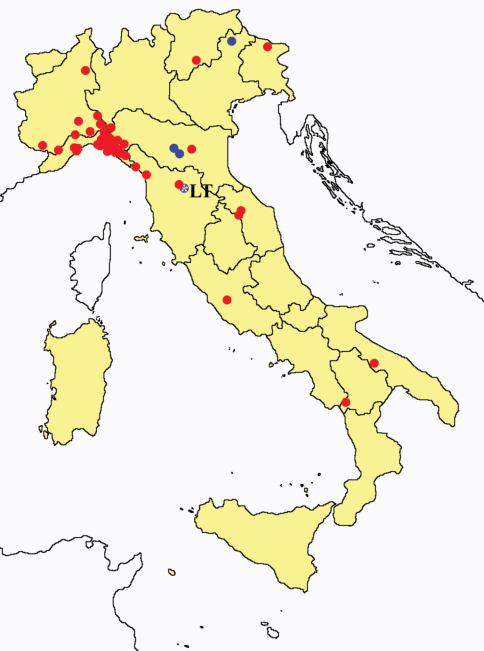
*Acerentulus confinis*: collecting sites in Italy (blue dots: data from literature; red dots: samples personally analyzed by the authors; LT = type area).

###### Remarks.

Bibliographic data from Berlese (1908), Nosek (1973), Fratello and Gioia (1975).

##### Acerentulus cunhai

Condé, 1950

http://species-id.net/wiki/Acerentulus_cunhai

Fig. 6 

###### Material examined.

1 ♀.

###### Distribution.

Central and West Europe, Macaronesia.

**Figure 6.**
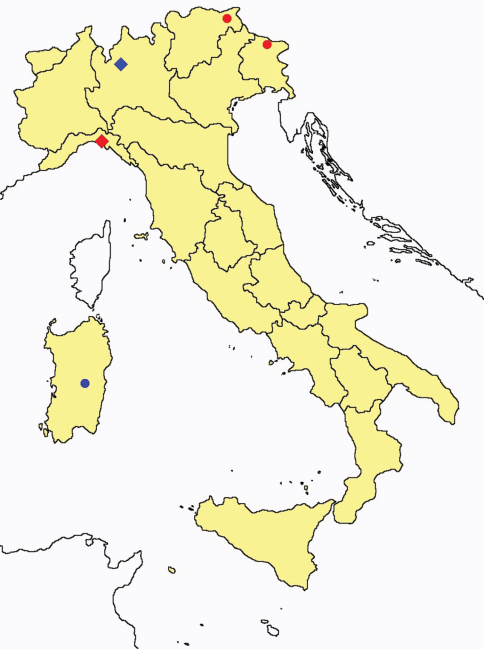
*Acerentulus* spp.: collecting sites in Italy (rhombus: *Acerentulus cunhai*; dots: *Acerentulus exiguus*; blue: data from literature; red: samples personally analyzed by the authors).

###### Remarks.

Bibliographic data from Dematteis (1971).

##### Acerentulus exiguus

Condé, 1944

http://species-id.net/wiki/Acerentulus_exiguus

Fig. 6 

###### Material examined.

1 ♂, 1 MJ.

###### Distribution.

Central and South Europe.

###### Remarks.

Bibliographic data from Dematteis Ravizza (1975).

##### Acerentulus gisini

Condé, 1952

http://species-id.net/wiki/Acerentulus_gisini

Fig. 7 

###### Material examined.

3 ♀♀.

###### Distribution.

Central Europe, Italy; data from Bulgaria should be confirmed (Szeptycki 2007).

**Figure 7.**
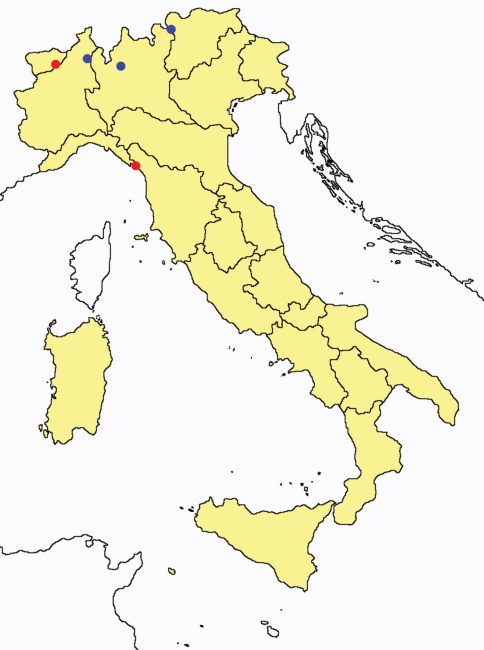
*Acerentulus gisini*: collecting sites in Italy (blue dots: data from literature; red dots: samples personally analyzed by the authors).

###### Remarks.

Bibliographic data from (Dematteis (1971, 1972).

##### Acerentulus terricola

Rusek, 1965

http://species-id.net/wiki/Acerentulus_terricola

Fig. 4 

###### Material examined.

2 ♂♂ (Bergeggi, Savona, Liguria).

###### Distribution.

Czech Republic (type area: Czech Rep., ‘Tal Suchý _leb im Nordteil des Mährischen Karstes“).

###### Remarks.

New record for the Italian fauna.

##### Acerentulus traegardhi

Ionesco, 1937

http://species-id.net/wiki/Acerentulus_traegardhi

Fig. 8 

###### Material examined.

15 ♂♂, 14 ♀♀, 5 PI, 16 MJ, 2 LII.

###### Distribution.

Recorded from nearly whole Europe, but it was commonly mistaken with *Acerentulus insignis*. Many data (especially from the West Europe) should be confirmed (Szeptycki 2007).

**Figure 8.**
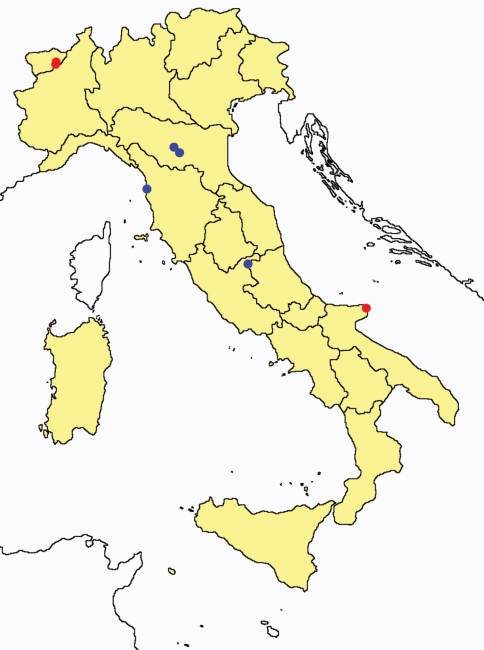
*Acerentulus traegardhi*: collecting sites in Italy (blue dots: data from literature; red dots: samples personally analyzed by the authors).

###### Remarks.

Bibliographic data from Nosek (1973), Fratello and Gioia (1975).

##### Acerentulus tuxeni

Rusek, 1966

http://species-id.net/wiki/Acerentulus_tuxeni

Fig. 4 

###### Material examined.

3 ♂♂, 3 ♀♀ (Ponte delle Alpi, Belluno, Veneto).

###### Distribution.

Central Europe.

###### Remarks.

New record for the Italian fauna.

##### Gracilentulus gracilis

(Berlese, 1908)

http://species-id.net/wiki/Gracilentulus_gracilis

Fig. 9 

###### Material examined.

3 ♂♂, 5 ♀♀.

###### Type area.

Tuscany, Toiana (Pisa).

###### Distribution.

Recorded from many European countries, from North Africa, South Africa, Australia and New Zealand.

**Figure 9.**
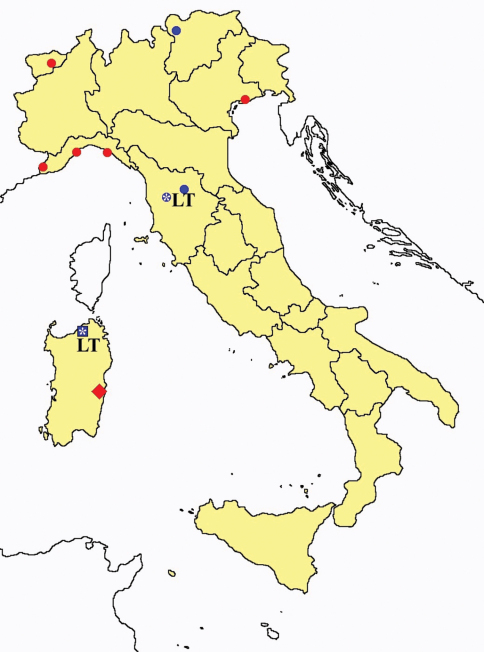
*Gracilentulus* spp.: collecting sites in Italy (dots: *Gracilentulus gracilis*; square: *Gracilentulus sardinianus*; rhombus: *Gracilentulus gracilis* + *Gracilentulus meridianus* + *Gracilentulus sardinianus*; blue: data from literature; red: samples personally analyzed by the authors; LT = type area).

###### Remarks.

Bibliographic data from Berlese (1908), Nosek (1973).

##### Gracilentulus meridianus

(Condé, 1945)

http://species-id.net/wiki/Gracilentulus_meridianus

Fig. 9 

###### Material examined.

4 ♂♂, 4 ♀♀ (Elini, Ogliastra, Sardinia).

###### Distribution.

France, Spain.

###### Remarks.

New record for the Italian fauna.

##### Gracilentulus sardinianus

Nosek, 1979

http://species-id.net/wiki/Gracilentulus_sardinianus

Fig. 9 

###### Material examined.

1 ♂, 3 ♀♀, 1 MJ.

###### Type area.

Sardinia, between Luogosanto and Tempio Pausania.

###### Distribution.

Type area only.

###### Remarks.

Bibliographic data from Nosek (1979).

##### Acerentomon affine

Bagnall, 1912

http://species-id.net/wiki/Acerentomon_affine

Fig. 10 

###### Material examined.

43 ♂♂, 63 ♀♀, 3 PI, 7 MJ, 1 undet.

###### Distribution.

West Europe; data from Romania and “Czechoslovakia” should be confirmed (Szeptycki 2007).

**Figure 10.**
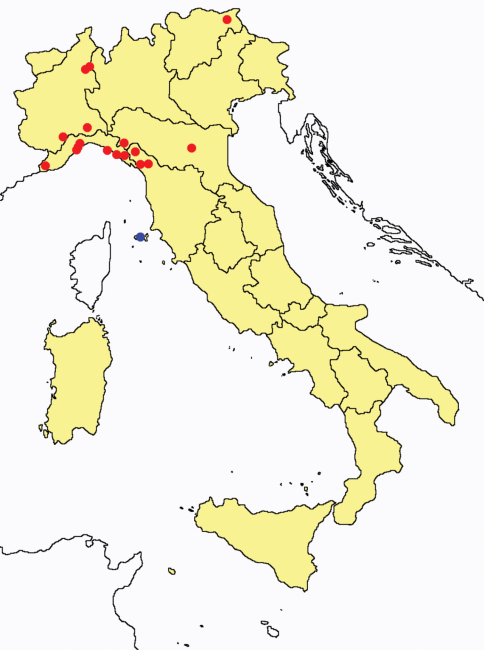
*Acerentomon affine*: collecting sites in Italy (blue dots: data from literature; red dots: samples personally analyzed by the authors).

###### Remarks.

Species confirmed for Italy. Bibliographic data from Fratello and Gioia (1975).

##### Acerentomon balcanicum

Ionesco, 1933

http://species-id.net/wiki/Acerentomon_balcanicum

Fig. 11 

###### Material examined.

17 ♂♂, 14 ♀♀, 1 PI, 1 MJ.

###### Distribution.

Southeast Europe, Ukraine.

**Figure 11.**
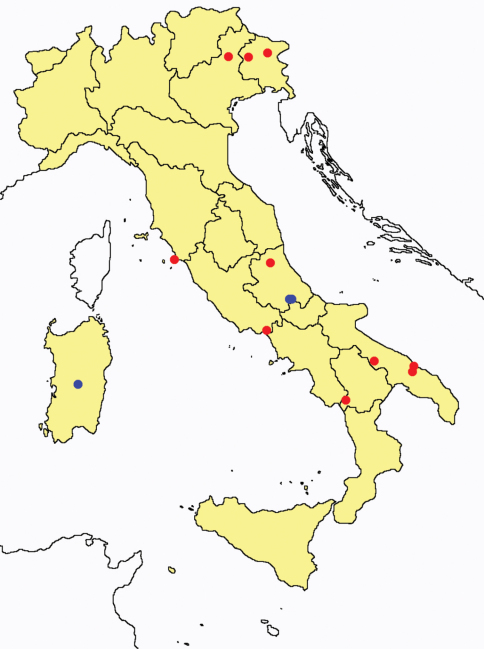
*Acerentomon balcanicum*: collecting sites in Italy (blue dots: data from literature; red dots: samples personally analyzed by the authors).

###### Remarks.

Bibliographic data from Nosek (1973).

##### Acerentomon baldense

Torti, 1986

http://species-id.net/wiki/Acerentomon_baldense

Fig. 18 

###### Material examined.

5 ♂♂, 7 ♀♀.

Type area: Veneto, Monte Balbo (Venetian PreAlps) surroundings of Prà Alpesina (Verona).

###### Distribution.

Type area only.

###### Remarks.

Bibliographic data from Torti (1986).

##### Acerentomon condei

Nosek & Dallai, 1982

http://species-id.net/wiki/Acerentomon_condei

Fig. 18 

###### Material examined.

6 ♂♂, 2 ♀♀, 1 MJ.

###### Type area.

Sardinia, Desulo (Gennargento).

###### Distribution.

Type area only.

###### Remarks.

Bibliographic data from Nosek and Dallai (1982).

##### Acerentomon doderoi

Silvestri, 1907

http://species-id.net/wiki/Acerentomon_doderoi

Fig. 12 

###### Material examined.

64 ♂♂, 94 ♀♀, 5 PI, 2 MJ.

###### Type area.

Liguria, Genoa.

###### Distribution.

Known only from Italy and Slovenia. All data from Central and West Europe and from USA are highly doubtful (Szeptycki 2007).

**Figure 12.**
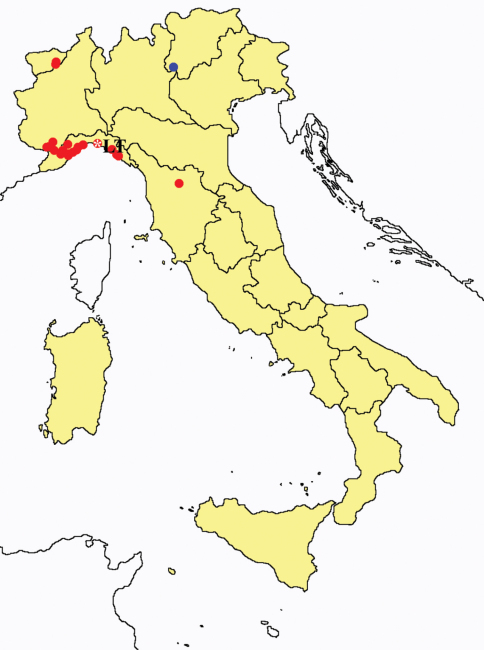
*Acerentomon doderoi*: collecting sites in Italy (blue dots: data from literature; red dots: samples personally analyzed by the authors; LT = type area).

###### Remarks.

We have not yet been able to analyse the type material from Villetta Dinegro (Genoa Town). Four specimens originally labelled as “cotypus *doderoi*” in Genoa Museum collection have been recently identified by the authors as *Acerentomon italicum*.

##### Acerentomon fageticola

Rusek, 1966

http://species-id.net/wiki/Acerentomon_fageticola

###### Distribution.

Central Europe.

###### Remarks.

Three specimens from Veneto (Cison, Treviso), and two from Liguria (Lavagna, Genoa) were identified by Prof. Nosek as *Acerentomon fageticola* and cited in a short note by Torti (1995a). These and some other similar specimens should be considered as individual variations of *Acerentomon italicum*: this hypothesis seems to be maintained by the coexistence in the same localities of individuals showing a continuum of diagnostic characters (foretarsal sensilla, chaetotaxy, pleural pectines) ranging from the *Acerentomon fageticola* to the *Acerentomon italicum* extremes, while we have not yet found sites where only “*fageticola-*type” specimens have been collected.

We hope that our current redescription of *Acerentomon italicum* could shed more light on the differences between this species and the related *Acerentomon fageticola*.

This species has been cited here and in the identification key only for exactness of information.

##### Acerentomon gallicum

Ionesco, 1933

http://species-id.net/wiki/Acerentomon_gallicum

Fig. 13 

###### Material examined.

42 ♂♂, 90 ♀♀, 7 PI, 11 MJ, 6 LII, 1 LI, 1 undet.

###### Distribution.

West and Central Europe, recorded also from Africa (Uganda – introduced?).

**Figure 13.**
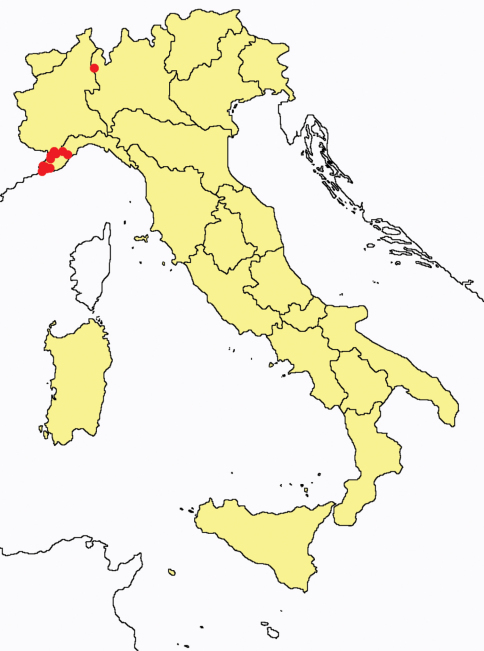
*Acerentomon gallicum*: collecting sites in Italy (red dots: samples personally analyzed by the authors).

###### Remarks.

Although in Szeptycki (2007) there is no information about the presence of this species in Italy, *Acerentomon gallicum* was cited in a short note by Torti (1995b).

##### Acerentomon italicum

Nosek, 1969

http://species-id.net/wiki/Acerentomon_italicum

Fig. 14 

###### Material examined.

433 ♂♂, 573 ♀♀, 18 PI, 16 MJ, 14 LII, 6 undet.

###### Type area.

Veneto, Colli Euganei near Padua, Italy.

###### Distribution.

Italy.

**Figure 14.**
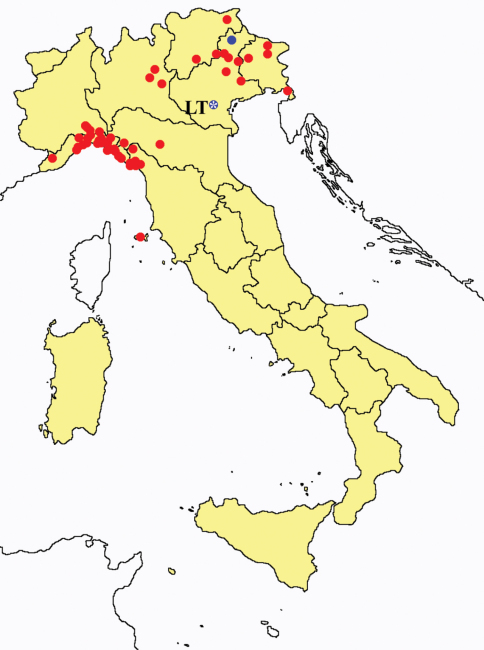
*Acerentomon italicum*: collecting sites in Italy (blue dots: data from literature; red dots: samples personally analyzed by the authors; LT = type area).

###### Remarks.

Species currently under redescription by the authors of this paper. Bibliographic data from (Nosek (1969, 1973).

##### Acerentomon maius

Berlese, 1908

http://species-id.net/wiki/Acerentomon_maius

Fig. 15 

###### Material examined.

353 ♂♂, 455 ♀♀, 40 PI, 25 MJ, 3 LII, 2 LI, 3 undet.

###### Type area.

Trentino Alto Adige, Tiarno.

###### Distribution.

Italy, Central Europe.

**Figure 15.**
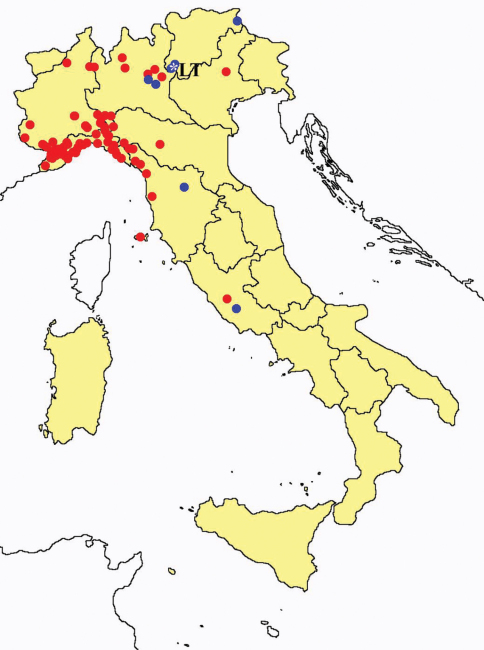
*Acerentomon maius*: collecting sites in Italy (blue dots: data from literature; red dots: samples personally analyzed by the authors; LT = type area).

###### Remarks.

Bibliographic data from Berlese (1908), Dematteis (1972), Nosek (1973), Fratello and Gioia (1975).

##### Acerentomon meridionale

Nosek, 1960

http://species-id.net/wiki/Acerentomon_meridionale

Fig. 16 

###### Material examined.

52 ♂♂, 93 ♀♀, 2 PI, 8 MJ, 1 undet.

###### Distribution.

South and Central Europe, Near East (Israel).

**Figure 16.**
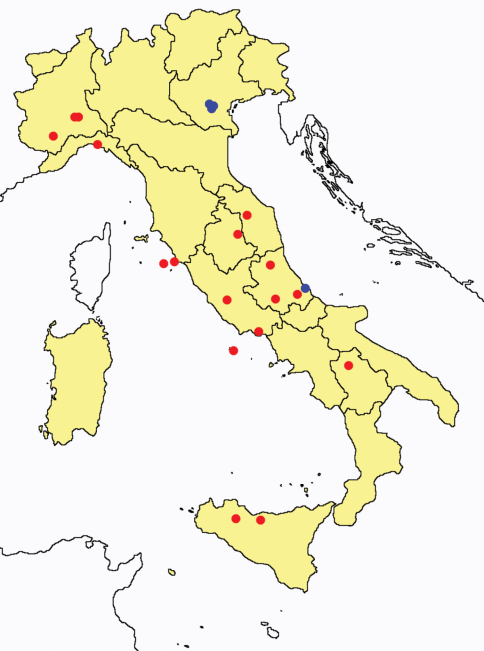
*Acerentomon meridionale*: collecting sites in Italy (blue dots: data from literature; red dots: samples personally analyzed by the authors).

###### Remarks.

Bibliographic data from Nosek (1973).

##### Acerentomon microrhinus

Berlese, 1909

http://species-id.net/wiki/Acerentomon_microrhinus

Fig. 17 

###### Material examined.

95 ♂♂, 161 ♀♀, 21 PI, 15 MJ, 1 LII, 2 undet.

###### Type area.

Piedmont, Casale Monferrato.

###### Distribution.

South and Central Europe.

**Figure 17.**
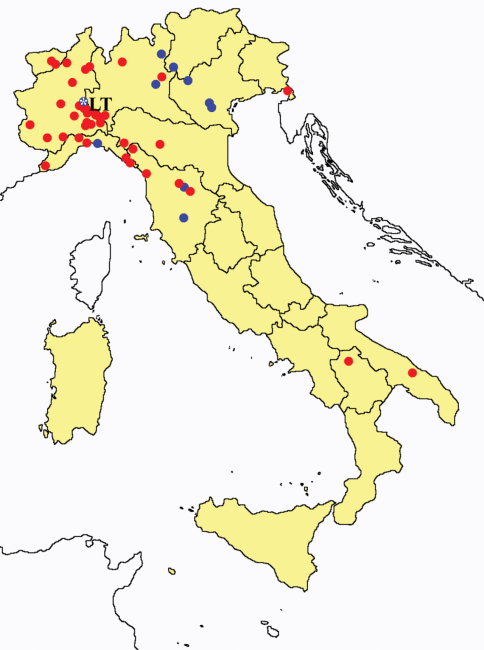
*Acerentomon microrhinus*: collecting sites in Italy (blue dots: data from literature; red dots: samples personally analyzed by the authors; LT = type area).

###### Remarks.

Bibliographic data from Berlese (1909), Dematteis (1972), Nosek (1973), Fratello and Gioia (1975).

##### Acerentomon noseki

Torti, 1981

http://species-id.net/wiki/Acerentomon_noseki

Fig. 18 

###### Material examined.

2 ♀♀.

###### Type area.

Piedmont, surroundings of Santuario di Oropa near Biella.

###### Distribution.

Type area only.

**Figure 18.**
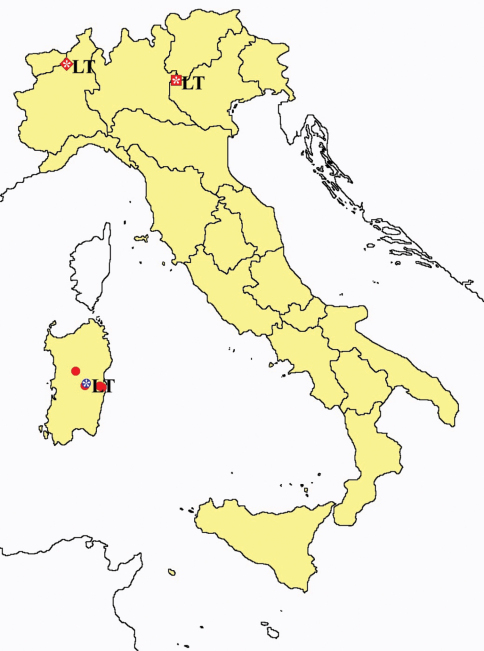
*Acerentomon* spp.: collecting sites in Italy (square: *Acerentomon baldense*; dots: *Acerentomon condei*; rhombus: *Acerentomon noseki*; blue: data from literature; red: samples personally analyzed by the authors; LT = type area).

###### Remarks.

Bibliographic data from Torti (1981a).

##### Acerella muscorum

(Ionesco, 1930)

http://species-id.net/wiki/Acerella_muscorum

Fig. 19 

###### Material examined.

3 ♂♂, 6 ♀♀.

###### Distribution.

Central and West Europe, Near East.

**Figure 19.**
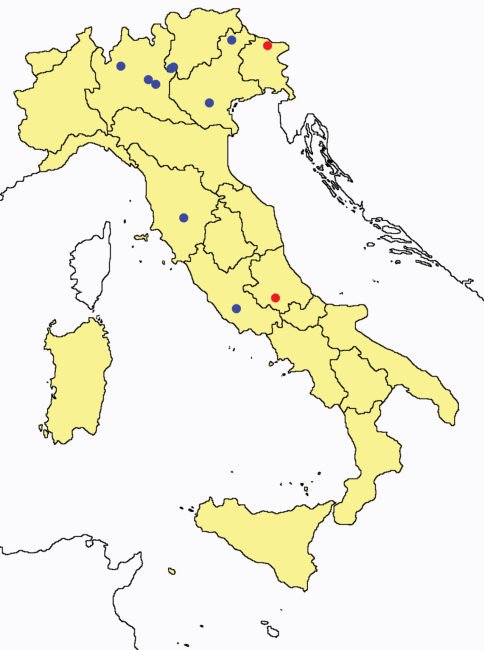
*Acerella muscorum*: collecting sites in Italy (blue dots: data from literature; red dots: samples personally analyzed by the authors).

###### Remarks.

Bibliographic data from Dematteis (1972), Nosek (1973), Fratello and Gioia (1975), Dallai et al. (2010).

##### Acerella tiarnea

(Berlese, 1908)

http://species-id.net/wiki/Acerella_tiarnea

Fig. 20 

###### Material examined.

30 ♂♂, 95 ♀♀, 1 PI, 1 MJ, 1 LI.

###### Type area.

Trentino Alto Adige, Tiarno.

###### Distribution.

Mediterranean Europe; all data from the Central and North Europe should be checked (Szeptycki 2007).

**Figure 20.**
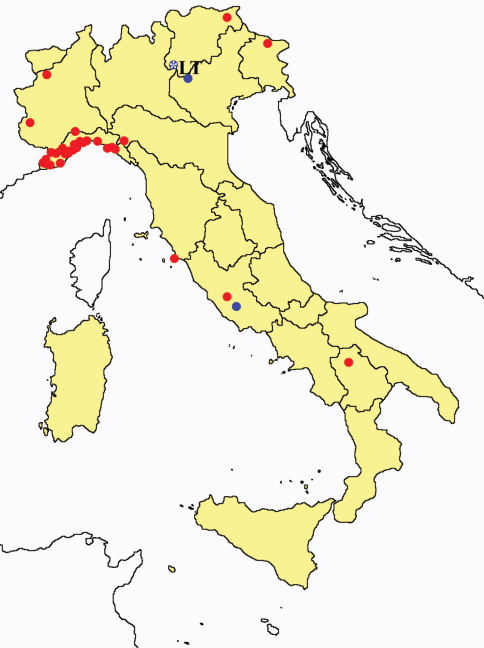
*Acerella tiarnea*: collecting sites in Italy (blue dots: data from literature; red dots: samples personally analyzed by the authors; LT = type area).

###### Remarks.

Bibliographic data from Berlese (1908), Dematteis (1972), Fratello and Gioia (1975).

### ORDO: EOSENTOMATA

#### Familia: Eosentomidae Berlese, 1909

##### Eosentomon armatum

Stach, 1926

http://species-id.net/wiki/Eosentomon_armatum

Fig. 21 

###### Material examined.

1 ♂ (Carlino, Udine, Friuli-Venezia Giulia) – 1 ♂, 6 ♀♀, 4 MJ (Floridia, Siracusa, Sicily).

###### Distribution.

Probably widely distributed in Europe, but all data before 1986 should be checked – they most likely concern not only *Eosentomon armatum*, but also some other similar species (Szeptycki 2007).

**Figure 21.**
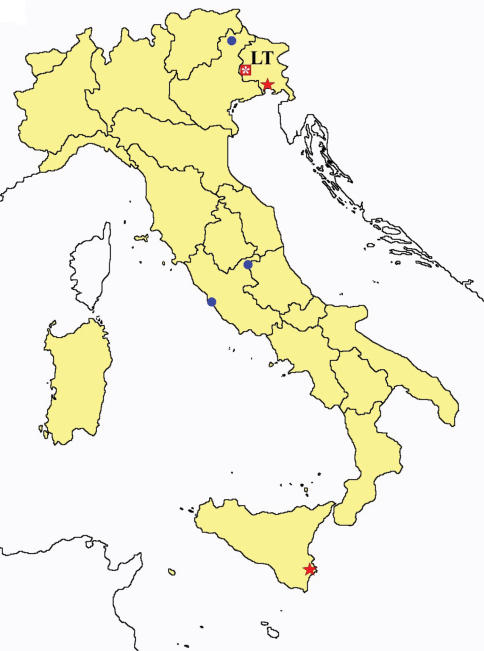
*Eosentomon* spp.: collecting sites in Italy (stars: *Eosentomon armatum*; square: *Eosentomon foroiuliense*; dots: *Eosentomon germanicum*; blue: data from literature; red: samples personally analyzed by the authors; LT = type area).

###### Remarks.

New record for the Italian fauna.

##### Eosentomon delicatum

Gisin 1945

http://species-id.net/wiki/Eosentomon_delicatum

Fig. 22 

###### Material examined.

8 ♂♂, 9 ♀♀, 2 MJ, 1 LII, 1 undet.

###### Distribution.

Europe, North Africa.

**Figure 22.**
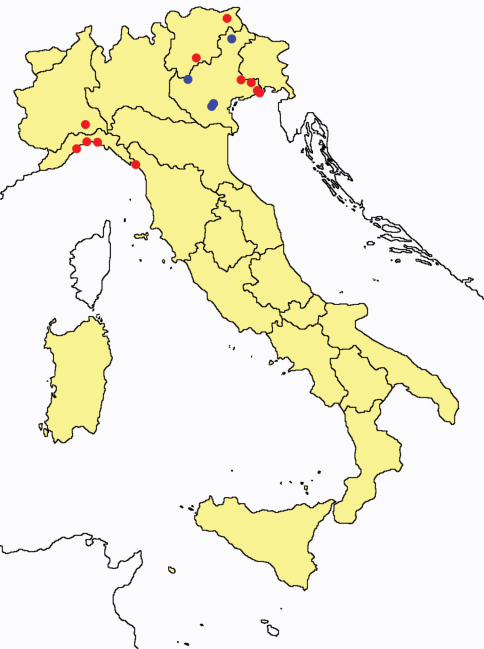
*Eosentomon delicatum*: collecting sites in Italy (blue dots: data from literature; red dots: samples personally analyzed by the authors).

###### Remarks.

Bibliographic data from Nosek (1973).

##### Eosentomon foroiuliense

Torti & Nosek, 1984

http://species-id.net/wiki/Eosentomon_foroiuliense

Fig. 21 

###### Material examined.

1 ♀.

###### Type area.

Friuli-Venezia Giulia, Aviano.

###### Distribution.

Type area only.

###### Remarks.

Bibliographic data from Torti and Nosek (1984).

##### Eosentomon germanicum

Prell, 1912

http://species-id.net/wiki/Eosentomon_germanicum

Fig. 21 

###### Distribution.

Central Europe, Scandinavia. The data from West Europe, Italy and Madeira (under *Eosentomon germanicum* and *Eosentomon forsslundi*) should be checked – *Eosentomon germanicum* was commonly mistaken with similar species (Szeptycki 2007).

###### Remarks.

Bibliographic data from Nosek (1973). We didn’t find specimens of this species in the collections we analyzed.

##### Eosentomon noseki

Tuxen, 1982

http://species-id.net/wiki/Eosentomon_noseki

Fig. 23 

###### Material examined.

43 ♂♂, 43 ♀♀, 1 PI, 18 MJ, 2 LII.

###### Distribution.

Macaronesia, Spain.

**Figure 23.**
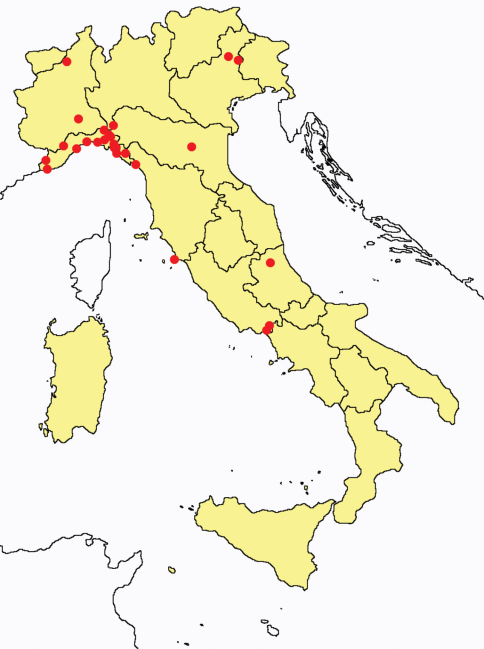
*Eosentomon noseki*: collecting sites in Italy (red dots: samples personally analyzed by the authors).

###### Remarks.

This species is not included in the World Catalogue (Szeptycki 2007) because it was recorded in Italy only more recently (Capurro et al. 2008a).

##### Eosentomon romanum

Nosek, 1969

http://species-id.net/wiki/Eosentomon_romanum

Fig. 24 

###### Type area.

Lazio, Rome.

###### Distribution.

Italy.

**Figure 24.**
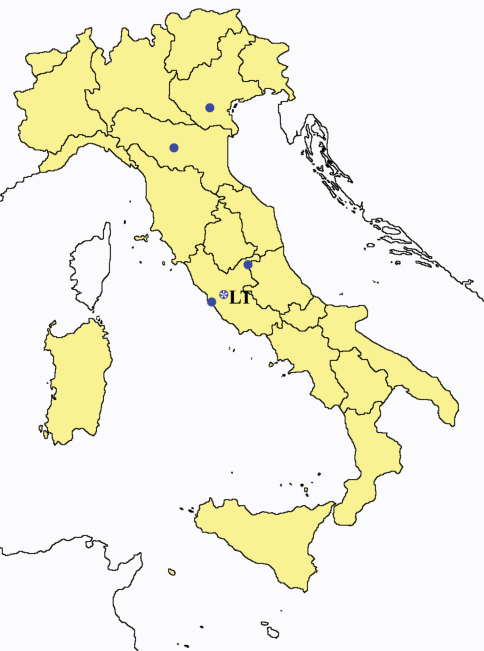
*Eosentomon romanum*: collecting sites in Italy (blue dots: data from literature; LT = type area).

###### Remarks.

Bibliographic data from Nosek (1969), Fratello and Gioia (1975).

##### Eosentomon transitorium

Berlese, 1908

http://species-id.net/wiki/Eosentomon_transitorium

Fig. 25 

###### Material examined.

112 ♂♂, 107 ♀♀, 38 MJ, 11 LII, 6 LI, 13 undet.

###### Type area.

Tuscany, Florence.

###### Distribution.

Probably whole Europe and North Africa, but most of the data should be confirmed (Szeptycki 2007).

**Figure 25.**
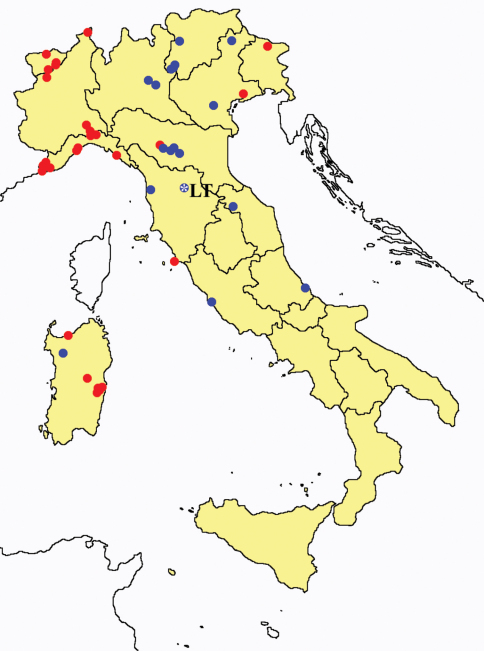
*Eosentomon transitorium*: collecting sites in Italy (blue dots: data from literature; red dots: samples personally analyzed by the authors; LT = type area).

###### Remarks.

Bibliographic data from Berlese (1908), Dematteis (1972), Nosek (1973), Dematteis Ravizza (1975), Fratello and Gioia (1975), Fratello and Sabatini (1989).

## Conclusion

**Figure 26.**
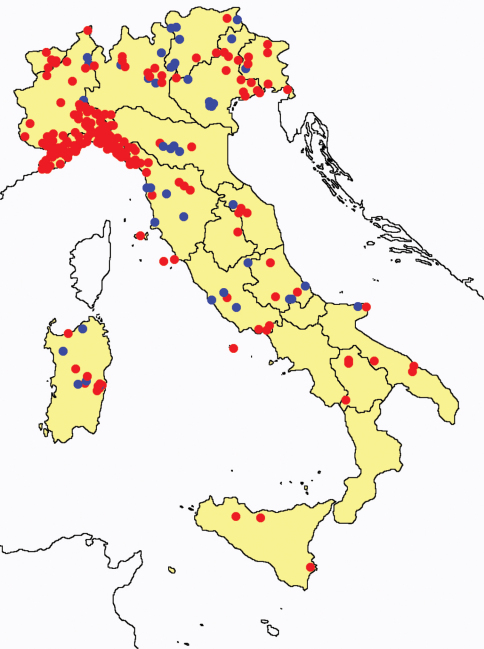
Distribution of the Protura sampling sites in Italy (blue dots: data known only from literature; red dots: data about specimens examined by the authors of this paper).

**Figure 27.**
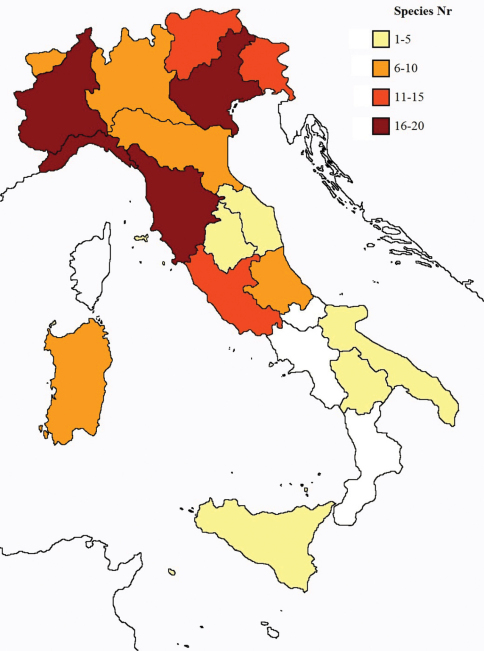
Species richness in the 20 Italian regions.

In Figures 26 and 27 the distribution of the sampling sites in Italy and the species richness in the Italian regions are shown, respectively. Unfortunately we regret for the lack of samples from Molise, Campania and Calabria (Southern part of the peninsula); but, apart from that, comparing maps on these Figures, it is clear that the species richness reflects the sampling effort in the different regions, with higher numbers of species known from regions such as Piedmont, Veneto and Liguria, where many more samples have been collected (for a detailed analysis of Protura of Liguria see Capurro et al. 2009).

According to the analysis made in this paper, we have been able to identify 40 Protura species in Italy, belonging to the families Hesperentomidae (1), Protentomidae (6), Acerentomidae (26) and Eosentomidae (7). At the species level, according to Vigna Taglianti et al. (1992), it is possible to outline the chorological categories shown in Table 2.

**Table 2.** Chorotypes of the Italian Protura.

**Chorotypes**	**Species nr**
Sub-Cosmopolitan	2
W-Palearctic	2
Turanic-European-Mediterranean	4
European-Mediterranean	5
European	3
Central-European	8
S-European	2
Mediterranean	4
Known only in Italy	10

Based on the findings, the Italian fauna is mainly composed of species having a European or Mediterranean distribution. With regard to the 10 species known only in Italy, it cannot be said to be endemic due to the poor level of knowledge of this taxon. For the same reason, that given in Table 2 should be considered only a preliminary attempt at classification, which, most likely, is susceptible to changes in the future.

The number of species and genera known in the European Countries (according to Szeptycki 2007; updated data for Austria and Ukraine are taken respectively from Christian 2011 and Shrubovych 2010) is shown in Table 3.

**Table 3.** Number of Protura species and genera in the European Countries.

**Country**	**Species nr**	**Genera nr**
Austria	58	10
Balearic islands	7	5
Belgium	4	3
Bosnia and Herzegovina	16	7
Bulgaria	4	2
Corsica	15	7
Croatia	4	2
Czech Republic	33	7
Denmark	7	5
Finland	3	2
France	38	10
Germany	44	10
Greece	13	9
Hungary	10	5
Iceland	2	1
Ireland	5	3
Italy	40	8
Lithuania	2	1
Luxemburg	30	10
Macedonia	2	2
The Netherlands	1	1
Norway	4	1
Poland	68	11
Portugal	15	5
Romania	10	5
Russia	7	4
Serbia	3	2
Slovakia	38	8
Slovenia	7	3
Spain	23	7
Sweden	12	5
Switzerland	11	6
Ukraine	58	12
United Kingdom	14	6

It seems rather unlikely that generally poorer (in terms of biodiversity) Countries such as Poland, Ukraine, Austria and Germany have more Protura species than Italy. It’s more likely that this gap is due to a lack of knowledge of the Italian fauna. In support of this hypothesis, a year spent on sampling project in a small cork oak wood in Liguria (NW Italy) led us to identify (Capurro et al. 2011) 11 species. We therefore assume that is extremely possible that other species distributed in neighbouring Countries – or Palearctic ones as well (see as is the case of *Acerentulus terricola*) – could be found in Italy.

We therefore hope that in the future we will be able to deepen and broaden our research to obtain a more accurate picture of Protura’s ecology and distribution.

## Supplementary Material

XML Treatment for Ionescuellum condei

XML Treatment for Protentomon berlesei

XML Treatment for Protentomon perpusillum

XML Treatment for Proturentomon condei

XML Treatment for Proturentomon minimum

XML Treatment for Proturentomon noseki

XML Treatment for Proturentomon pilosum

XML Treatment for Acerentulus alpinus

XML Treatment for Acerentulus apuliacus

XML Treatment for Acerentulus condei

XML Treatment for Acerentulus confinis

XML Treatment for Acerentulus cunhai

XML Treatment for Acerentulus exiguus

XML Treatment for Acerentulus gisini

XML Treatment for Acerentulus terricola

XML Treatment for Acerentulus traegardhi

XML Treatment for Acerentulus tuxeni

XML Treatment for Gracilentulus gracilis

XML Treatment for Gracilentulus meridianus

XML Treatment for Gracilentulus sardinianus

XML Treatment for Acerentomon affine

XML Treatment for Acerentomon balcanicum

XML Treatment for Acerentomon baldense

XML Treatment for Acerentomon condei

XML Treatment for Acerentomon doderoi

XML Treatment for Acerentomon fageticola

XML Treatment for Acerentomon gallicum

XML Treatment for Acerentomon italicum

XML Treatment for Acerentomon maius

XML Treatment for Acerentomon meridionale

XML Treatment for Acerentomon microrhinus

XML Treatment for Acerentomon noseki

XML Treatment for Acerella muscorum

XML Treatment for Acerella tiarnea

XML Treatment for Eosentomon armatum

XML Treatment for Eosentomon delicatum

XML Treatment for Eosentomon foroiuliense

XML Treatment for Eosentomon germanicum

XML Treatment for Eosentomon noseki

XML Treatment for Eosentomon romanum

XML Treatment for Eosentomon transitorium
